# Efficacy and safety of levodopa–carbidopa intestinal gel from a study in Japanese, Taiwanese, and Korean advanced Parkinson’s disease patients

**DOI:** 10.1038/npjparkd.2016.20

**Published:** 2016-11-03

**Authors:** Miho Murata, Masahito Mihara, Kazuko Hasegawa, Beomseok Jeon, Chon-Haw Tsai, Noriko Nishikawa, Tomoko Oeda, Masayuki Yokoyama, Weining Z Robieson, Davis Ryman, Susan Eaton, Krai Chatamra, Janet Benesh

**Affiliations:** 1 National Center of Neurology and Psychiatry, Tokyo, Japan; 2 Osaka University Hospital, Suita, Japan; 3 National Hospital Organization Sagamihara National Hospital, Sagamihara, Japan; 4 Seoul National University Hospital, Seoul, Korea; 5 Department of Neurology, China Medical University Hospital and Medical College, China Medical University, Taichung, Taiwan; 6 Ehime University Hospital, Matsuyama, Japan; 7 National Hospital Organization Utano Hospital, Kyoto, Japan; 8 AbbVie Inc, Tokyo, Japan; 9 AbbVie Inc, North Chicago, IL, USA

## Abstract

In a previous multinational, randomized, double-blind, double-dummy study, levodopa–carbidopa intestinal gel (LCIG) was tolerable and significantly improved ‘off’ time in advanced Parkinson’s disease (PD) patients. However, efficacy and safety in the Asian population has not yet been demonstrated. In this open-label study, efficacy and safety of LCIG were assessed in Japanese, Korean, and Taiwanese advanced PD patients with motor complications not adequately controlled by available PD medication. The patients were treated with LCIG monotherapy for 12 weeks. The primary end point was the mean change from baseline to week 12 in ‘off’ time, as reported in the PD Symptom Diary, normalized to a 16 h waking day and analyzed by a mixed-model repeated-measures analysis. Adverse events (AEs) were recorded. Thirty-one patients were enrolled (23 Japanese, 4 Taiwanese, 4 Korean) and 28 (90%) completed the study. For those who completed the study, the mean (s.d.) total daily levodopa dose from LCIG was 1,206.3 (493.6) mg/day at final visit (*n*=28); last observation carried forward (*n*=30) was 1,227.6 (482.8) mg/day. There was a significant mean change (s.d.) of −4.6 (3.0) hours of ‘off’ time from baseline (mean (s.d.)=7.4 (2.3)) to week 12 (*n*=29), *P*<0.001. All the patients had an AE, with the most frequently reported being incision site pain (42%); 1 (3.2%) discontinued treatment because of an AE and later died because of sepsis, which the investigator considered unrelated to LCIG treatment. These results suggest that LCIG is efficacious and tolerable in Japanese, Taiwanese, and Korean advanced PD patients.

## Introduction

Although oral levodopa is the primary treatment for Parkinson’s disease (PD), prolonged use is associated with the development of motor complications, such as dyskinesias and ‘on’/‘off’ fluctuations, that can often be problematic with advancing disease.^1,2^ These motor complications are due, in part, to a continual loss of dopaminergic neurons, as well as effects of the short half-life of levodopa and gastric emptying leading to non-physiologic pulsatile stimulation of striatal dopamine receptors.^3,4^ As PD progresses, the therapeutic window narrows, and there is an increase in motor complications associated with high and low plasma levodopa concentrations from frequent, intermittent exposure to oral levodopa.^4–7^

Levodopa–carbidopa intestinal gel (LCIG; known in Japan as ABT-SLV187, and in the United States as carbidopa–levodopa enteral suspension) provides continuous levodopa infusion directly into the proximal small intestine via percutaneous endoscopic gastrojejunostomy (PEG-J), and reduces fluctuations in plasma concentrations of levodopa.^8,9^ In a prior 12-week, double-blind, double-dummy clinical trial evaluating the efficacy and safety of LCIG in advanced PD patients, LCIG treatment reduced the mean ‘off’ time by 4.04 h per day from baseline to final, which was significant compared with oral levodopa–carbidopa immediate release (LC-IR) therapy (mean change=−2.14 h per day).^10^ In that double-blind study and additional open-label studies, LCIG was well tolerated.^10–12^ Safety data from these studies were integrated, and the overall rate of discontinuations due to adverse events (AEs) was 17% of patients over a median exposure period of 911 days.^13^ Although these studies were multinational, the advanced PD patient populations were primarily (at least 92%) Caucasian.^1,11,12^

Similar to Caucasian populations, cross-sectional and retrospective studies have confirmed that motor fluctuations are prevalent in Asian PD patients.^14–18^ However, Asian PD patients develop dyskinesias more frequently than Caucasians,^19,20^ and two retrospective studies indicated that Japanese females have a shorter onset of wearing-off and dyskinesia than Japanese males.^17,18^ Asian PD patients have required 20–30% lower doses of oral levodopa to control PD symptoms,^19,20^ which may reflect a difference in BMI and under treatment of Asian PD patients overall. Japanese patients metabolized carbidopa at a lower rate than Caucasian patients.^9^ However, a pharmacokinetic study showed that continuous LCIG infusion led to lower plasma levodopa fluctuations and a reduction in motor fluctuations than oral levodopa in Japanese advanced PD patients (*n*=4).^8,9^ The efficacy and safety data of LCIG in Asian patients remains limited.^9^ The main objective of this open-label study was to assess the efficacy and safety of LCIG monotherapy in Japanese, Taiwanese, and Korean patients with advanced idiopathic PD and persistent motor complications despite optimized treatment with available oral anti-Parkinsonian medications.

## Results

**Figure 1:**
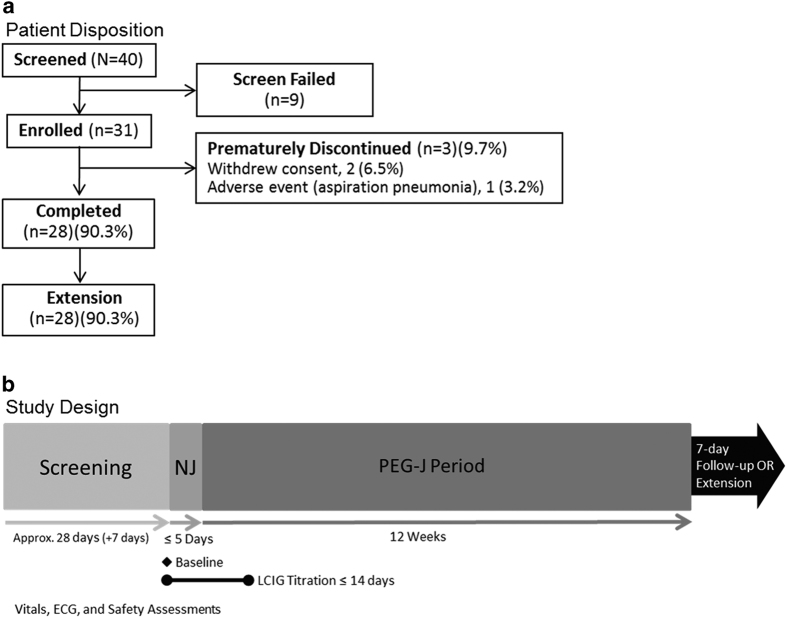
(**a**) Patient disposition and (**b**) study design. The NJ period was expected to be 5 days, but could vary. Patients who prematurely discontinued the study and did not continue into the separate extension study had a follow-up visit 7 days after discontinuation. NJ, nasojejunum; PEG-J, percutaneous endoscopic gastronomy-jejunum.

There were 31 patients enrolled (Figure 1a), including 23 in Japan, 4 in Taiwan, and 4 in Korea. Of these 31 patients, 30 entered the PEG-J period (Figure 1b), and all the 28 patients (90.3%) who completed the study continued into the ongoing extension study. Before the study, all 31 patients (100%) were on at least one levodopa-containing PD medication (levodopa, Madopar, or Sinemet/Menesit; Supplementary Table 1). Following the collection of baseline assessments (Table 1), all the 31 patients tapered their PD medication before nasojejunum (NJ) placement. During the study, the most common concomitant medications were routine medications given for a PEG-J procedure (Supplementary Table 1).

The patients reached a stable dose of LCIG in a mean (s.d.) 5.6 (2.6) days (*n*=30). On the basis of the dosing diary of patients who completed the study, the mean (s.d.) total daily levodopa dose from LCIG was 1,206.3 (493.6) mg/day (*n*=28) at final visit, and for all the patients (*n*=30), 1,227.6 (482.8) mg/day. The median (range) time of patient exposure to LCIG was 6.9 (3–14) days during the NJ period (*N*=31) and 81.5 (18–87) days during the PEG-J period (*n*=30).

### Efficacy

Among the patients who had post-PEG-J PD diary assessments, one patient did not maintain LCIG monotherapy and started concomitant medication of rotigotine and levodopa on treatment day 60. This patient’s PD diary assessments completed before this day were included in the primary efficacy analysis of ‘off’ time and those recorded afterwards were excluded.

**Figure 2:**
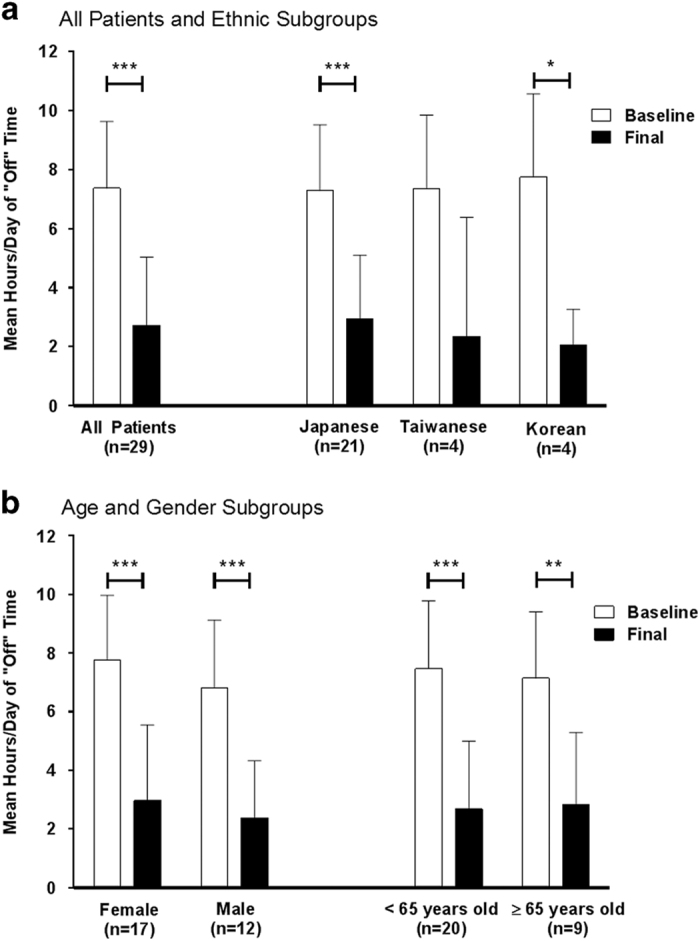
Mean daily hours of ‘off’ time at baseline and final visit in (**a**) all patients and ethnic subgroups and (**b**) age and gender subgroups. Daily totals were normalized to a 16 h waking day and the 3 days before the visit were averaged. Error bars indicate standard deviation. *P* values from a two-sided one-sample *t*-test indicate statistically significant mean change from baseline of *P⩽0.05, **P⩽0.01, and ***P⩽0.001.

**Figure 3:**
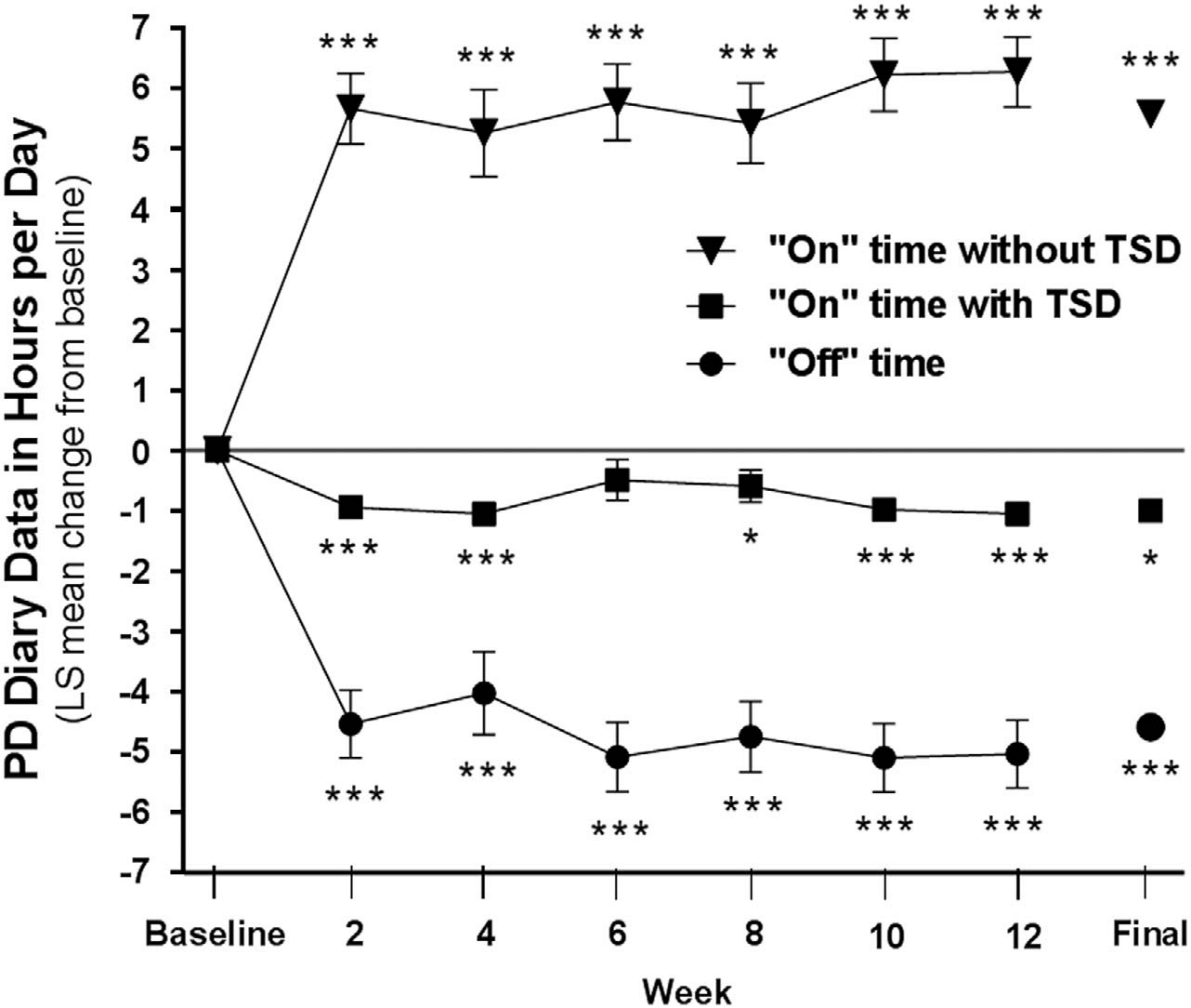
Change from baseline for PD diary measures. Daily totals were normalized to a 16 h waking day and the 3 days before the visit were averaged. ‘On’ time without troublesome dyskinesia is the sum of ‘on’ time without dyskinesia and ‘on’ time with non-troublesome dyskinesia. At baseline, the mean (s.d.) daily hours of PD Symptom Diary measures were: ‘off’ time=7.4 (2.3), ‘on’ time with TSD=1.1 (2.3), and ‘on’ time without TSD=7.5 (2.5). Error bars indicate standard error. *P* values indicate statistically significant mean change from baseline of *P⩽0.05 and ***P⩽0.001. At baseline, weeks 1–6, and final, the sample size was 29; and at week 8, it was 27 for all the measures. At weeks 10 and 12, the sample size was 28 for ‘on’ time with and without TSD and 27 for ‘off’ time. PD, Parkinson's disease; TSD, troublesome dyskinesia.

At week 12 (Figure 2a), the mean (s.d.) hours of ‘off’ time was significantly reduced by 4.6 (3.0) hours per day, compared with baseline, *P*<0.001. The improvement in ‘off’ time was observed in Japanese (*n*=21), Korean (*n*=4), and Taiwanese (*n*=4) subgroups (Figure 2a). The LCIG treatment led to significant improvements in ‘off’ time across all gender and age subgroups (Figure 2b). Significant improvements in ‘off’ time, and ‘on’ time with and without troublesome dyskinesia (TSD) were observed in the study population as early as week 2, and persisted for the remainder of the study (Figure 3).

**Figure 4:**
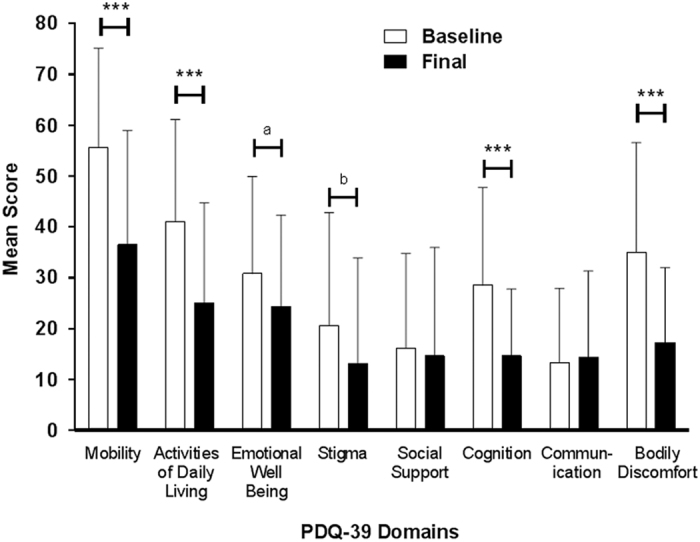
Mean PDQ-39 domain scores at baseline and final visit. *N*=30. Error bars indicate standard deviation. *P* values from a two-sided one-sample *t*-test indicate statistically significant mean change from baseline; ***P⩽0.001, ^a^*P*=0.059, ^b^*P*=0.070. PDQ, Parkinson’s Disease Questionnaire.

At the final visit, there was a significant improvement in the quality of life, based on the mean change from baseline of the 39-item Parkinson’s Disease Questionnaire (PDQ-39) summary index of −12.0 (11.5), *P*<0.001 (*n*=30). Notably, the quality of life significantly improved in the mobility, activities of daily living, cognition, and bodily discomfort domains of the PDQ-39 (Figure 4). At week 12, the majority of patients (23/29, 79.3%) rated their change in the quality of life on the Patient Global Impression of Change as ‘much improved’ or ‘very much improved’ (Supplementary Table 2), and the mean (s.d.) score 1.9 (0.8) was significantly different from a hypothesized mean score of 4 (no change), *P*<0.001. The results were similar on the Clinical Global Impression of Change, rated by investigators (Supplementary Table 2) and the mean (s.d.) score of 2.0 (0.9) was significantly different from a hypothesized mean score of 4 (no change), *P*<0.001.

Hierarchical testing of secondary efficacy measures ceased after there was no statistically significant mean (s.d.) change from baseline to final visit on the Unified Parkinson’s Disease Rating Scale (UPDRS) Part II (−1.8 (5.8), *P*=0.101).

### Safety

All the patients had an AE, and the most frequently reported AEs were associated with the gastrointestinal tract or procedure (Table 2). Most of the reported AEs were mild or moderate in severity as rated by the study investigator. Most AEs, including procedure or device associated AEs, occurred within the first week following the PEG-J placement procedure and resolved (Supplementary Figure 1).

Four patients (12.9%) had at least one serious AE (Table 2). Of these four patients, two had events that the study investigator considered to have a reasonable possibility of being related to the treatment system (drug/device): one had severe pneumonia aspiration, device (J-tube) kink, device (J-tube) dislocation, and gastrointestinal perforation, and the other patient had moderate abdominal pain and constipation. Both the patients recovered.

Of the four patients who had one or more serious AE, two had serious AEs that were considered unrelated to the treatment system; one had melena on day 11 and later recovered; and the other was an 83-year-old female who had a femur fracture on day 25 (last LCIG dose on day 26), developed severe pneumonia aspiration on day 62, severe sepsis on day 64, and disseminated intravascular coagulation on day 65. She died because of sepsis on day 86; investigators did not consider the death (one patient, 3.2%) to have a reasonable possibility of being related to LCIG treatment. One patient (the latter, 3.2%) discontinued the study because of AEs (pneumonia and sepsis). No other patients prematurely discontinued or died during the study or follow-up period. There were no clinically meaningful changes in vitals, electrocardiograms, and laboratory values compared with the baseline.

No clinically meaningful differences in the safety profile of LCIG occurred between ethnic subgroups. The safety profile of LCIG in Japanese patients was similar to that of the overall study population, and the AEs occurring in more than 15% of Japanese patients (*n*=23) were incision site pain (52%), excessive granulation tissue (35%), fall (22%), nasopharyngitis (22%), constipation (17%), diarrhea (17%), and procedural pain (17%). The AEs occurring in at least two Taiwanese patients (*n*=4) were increased homocysteine (50%) and constipation (50%), and in at least two Korean patients (*n*=4) were abdominal pain (50%), diarrhea (50%), and pyrexia (50%).

## Discussion

The treatment options are limited for Japanese advanced PD patients.^21^ Deep brain stimulation therapy has been the primary treatment option for advanced PD patients in Japan since 2000, nearly replacing stereotactic ablative surgical therapy.^21^ LCIG is a long-term treatment option for advanced PD patients with motor complications that persist despite optimized treatment with available oral anti-Parkinsonian medications, and has been commercially available in Europe since 2004. Although a previous pharmacokinetic study of LCIG in Japanese patients has shown that continuous infusion of levodopa results in reduced fluctuation index,^9^ this is the first study to assess the efficacy and safety of LCIG in a larger Asian population. In this prospective open-label study, Asian advanced PD patients with motor complications not adequately controlled by available anti-Parkinsonian medication were enrolled. In this patient population, LCIG infusion replaced oral levodopa and other anti-Parkinsonian medication.

At most time points, continuous LCIG infusion led to a significant improvement in ‘off’ time without an increase in ‘on’ time with TSD. The improvements in motor fluctuations were consistent with a prior 12-week double-blind study and 54-week open-label study, in which the majority of advanced PD patients were Caucasian and showed significant improvements in ‘off’ time and ‘on’ time without TSD within the first 12 weeks of LCIG treatment.^12^

The improvement in ‘off’ time was, in general, independent of gender, age, and ethnicity. There were no clinically meaningful differences in ‘off’ time between Japanese, Korean, and Taiwanese advanced PD patients. Although the reduction in ‘off’ time in Taiwanese PD patients was not statistically significant, the sample size was small.

The improvements in motor symptoms coincided with improvement in the quality of life in Asian PD patients. The decrease (improvement) in the UPDRS Part II score was not statistically significant, which was inconsistent with the significant improvements in the activities of daily living domain of the PDQ-39 and PDQ-39 summary index. However, responses on the Patient Global Impression of Change indicated that the majority of patients believed they had improved quality of life at the end of the study, which supported the PDQ-39 results. Clinician-rated Clinical Global Impression of Change responses further demonstrated that all but one patient had improved quality of life.

The safety profile of LCIG in Asian advanced PD patients is consistent with previous open-label studies in which the majority of advanced PD patients had an AE.^11,12^ The most common AEs were primarily related to the procedure or gastrointestinal tract, mild to moderate in severity, and known complications of the PEG-J placement procedure.^22,23^ Some gastrointestinal procedure AEs, such as gastrointestinal perforation, can be life-threatening; however, all serious AEs in this study that were considered to be possibly related to LCIG treatment were resolved. The most common AEs that were not related to the procedure or gastrointestinal tract are associated with levodopa exposure (dyskinesia, increased blood homocysteine), underlying PD (fall, dyskinesia), and the elderly population.

Although all patients had an AE, the most common AEs that occurred in each ethnic subgroup varied. It is very likely that this variation is because of the imbalance in sample size among the ethnic subgroups, as none of the variations were considered clinically meaningful. This study was limited by the relatively small sample size, particularly in the Korean and Taiwanese patients. The study was open-label and only levodopa-responsive patients were included, which may have introduced selection bias to the outcomes.

Despite the high incidence of AEs in this study, the discontinuation rate because of AEs (3.2%) was low and similar to a double-blind study with a similar sample size and duration, which supports the overall tolerability of LCIG in Asian advanced PD patients. This is the first study to demonstrate safety and efficacy of LCIG in Asian advanced PD patients, and further substantiates the robust and consistent nature of LCIG treatment in the general advanced PD population.^,11,12^

## Materials and Methods

The efficacy and safety of LCIG were evaluated in a Phase 3, 12-week, single-arm, open-label, baseline-controlled, multicenter study in Japan, South Korea, and Taiwan (NCT01960842/JapicCTI-142431) from October 2013 to March 2015. The study protocol was approved by the institutional review board/ethics committee at all the 13 centers in the three countries. All the patients provided written, informed consent.

### Study design

The study consisted of four time periods: screening (up to 35 days), titration of LCIG via NJ tube (AbbVie, North Chicago, IL, USA and Covidien/Medtronic, Minneapolis, MN, USA), infusion via PEG-J for 12 weeks, and a 7-day follow-up period (Figure 1b). During screening, the patients were converted to LC-IR (levodopa–carbidopa 100 mg/10 mg IR tablets (MSD K K, Tokyo, Japan) or levodopa–carbidopa 100 mg/25 mg IR tablets (Sun Pharmaceuticals Industries, Mumbai, India)) as a monotherapy before the NJ procedure. The patients who completed this study and continued onto the separate extension study did not complete the 7-day follow-up period.

The LCIG system (Duodopa; designated in the United States as carbidopa–levodopa enteral suspension/Duopa) includes an infusion pump (CADD Legacy 1400, Smiths Medical, Ashford, Kent, UK) and a 100 ml medication cassette (Fresenius Kabi, Bad Homburg, Germany; and AbbVie) worn outside the body, connected to the inner jejunal extension tube (FR9, Fresenius Kabi) via PEG-tubing (FR15, Fresenius Kabi). LCIG (20 mg/ml levodopa and 5 mg/ml carbidopa) monotherapy was administered during 16 waking hours as a morning dose, continuous maintenance dose, and intermittent extra doses. The patients had the option to take LC-IR at night.

### Patients

The patients were at least 30 years old, levodopa-responsive, and diagnosed with idiopathic PD according to the United Kingdom Parkinson’s Disease Society Brain Bank Criteria. The patients had severe motor fluctuations with at least 3 h of ‘off’ time per day at baseline despite individually optimized PD therapy and recognizable ‘off’ and ‘on’ mobility states, as observed by study investigator and confirmed by PD diary records.^24^ The patients with secondary Parkinsonism, Parkinson’s plus syndromes, other neurodegenerative diseases, neurological deficits, or those who had undergone neurosurgery were ineligible.

### Efficacy

The patients recorded the number of hours with ‘off’ time, ‘on’ time without dyskinesia, ‘on’ time with non-TSD, and ‘on’ time with TSD in an at-home PD Symptom Diary.^24^ The primary efficacy measure was the change from baseline to final in the mean daily ‘off’ time. Secondary measures included (in hierarchical order of testing): daily ‘on’ time without TSD (sum of ‘on’ time without dyskinesia and ‘on’ time with non-TSD), Parkinson’s Disease Questionnaire-39 (PDQ-39) summary index,^25^ Clinical Global Impression of Change score, Patient Global Impression of Change score, UPDRS total score,^26^ UPDRS Parts II and III scores, daily ‘on’ time with TSD, PDQ-39 domain scores,^25^ and UPDRS Parts I and IV scores. The change from baseline in mean daily ‘off’ time at weeks 2, 4, 6, 8, 10, and 12 was also evaluated. During the treatment period, the UPDRS assessments were done during ‘on’ time.

### Safety

The treatment-emergent AEs reported here include all AEs with onset on or after the date of PEG placement and within 7 days of the end of LCIG treatment. The AEs were coded using the MedDRA (Medical Dictionary for Regulatory Activities) and were tabulated by MedDRA preferred term version 17.1.^27^ The local study investigators rated the severity of each AE (mild, moderate, severe), the seriousness (serious or non-serious^28^), and whether or not there was a reasonable possibility that the LCIG system (drug/device) had a causal relationship with the AE. The gastrointestinal AEs of special interest (AESIs) were tabulated by the Standardized MedDRA Query for gastrointestinal- and gastrointestinal procedure-related events.^27^ The clinical laboratory, electrocardiogram, and vital signs were collected throughout the study.

### Statistical analysis

The sample size of 21 patients was required to provide at least 90% power for the primary efficacy end point at a two-sided statistical significance level of 0.05, assuming that the improvement in ‘off’ time in Asian subjects after 12 weeks of treatment was 3.9 h with a standard deviation of 4 h. Total enrollment of 32 was determined on the basis of the assumption that 20% of patients would not have post-baseline PD Diary assessments or prematurely discontinue.

The baseline was defined as the last non-missing observation before the patients converted to LC-IR monotherapy. The final was defined as the last non-missing observation that was no more than 1 day after the last infusion of LCIG. The daily levodopa dose included the morning, continuous maintenance, and extra doses of LCIG, but not LC-IR taken at night.

The safety was analyzed in all the patients who underwent the NJ placement procedure. The efficacy was analyzed in patients who received at least one dose of LCIG via PEG-J with at least one post-baseline measurement of at least one efficacy measure.

Daily totals in the PD Diary were normalized to 16 h waking hours and averaged for the 3 days before the visit. The primary end point in all the patients and subgroups defined by ethnicity were analyzed using a one-sample, two-sided *t*-test. After statistical significance (*P*⩽0.050) was demonstrated on the primary end point, secondary measures were tested in a hierarchical order (see efficacy section) until the analysis failed to demonstrate statistical significance. For the majority of secondary efficacy measures, the change from baseline to each time point was evaluated by a mixed-model repeated-measures analysis, which included fixed effects of ethnicity and visit, baseline value as a covariate, and baseline×visit interaction. The Clinical Global Impression of Change and Patient Global Impression of Change scores were tested with a two-sided, one-sample Wilcoxon signed-rank test compared with the null hypothesis of a mean score of 4 (no change).

## Figures and Tables

**Table 1:** Patients’ baseline characteristics

*Characteristic*	*Number of patients (%)*	*Mean±s.d. [range]*
Age, years		61.6±10.5 [45.0–83.0]
		
*Sex*		
Female	19 (61)	
Male	12 (39)	
		
*Ethnicity*		
Japanese	23 (74)	
Taiwanese	4 (13)	
Korean	4 (13)	
Duration of PD, years		12.4±5.1 [2.9–29.2]
Levodopa dose,^a^ mg/day	30 (97)	1,011.7±629.7
‘Off’ time, hours/day	29 (94)	7.4±2.3 [3.0–11.6]
‘On’ time without troublesome dyskinesia, hours/day	29 (94)	7.5±2.5 [1.0–11.8]
‘On’ time with troublesome dyskinesia, hours/day	29 (94)	1.1±2.3 [0.0–9.2]
PDQ-39 summary index	30 (97)	35.5±13.8 [8–67]
UPDRS total score	30 (97)	27.7±15.5 [2–60]
UPDRS Part II score	30 (97)	9.4±6.6 [0–24]
UPDRS Part III score	30 (97)	16.5±9.7 [1–42]

Abbreviations: PDQ, Parkinson’s Disease Questionnaire; UPDRS, Unified Parkinson’s Disease Rating Scale.

*N*=31 except as noted. Daily hours of ‘off’ time and ‘on’ time with/without troublesome dyskinesia were recorded in the PD Symptom Diary, normalized to a 16 h waking day and averaged for the 3 days before the visit. ‘On’ time without troublesome dyskinesia is the sum of ‘on’ time without dyskinesia and ‘on’ time with non-troublesome dyskinesia.

a Last full daily levodopa dose of levodopa–carbidopa immediate release tablets before the NJ procedure.

**Table 2:** Summary of adverse events (AEs) and serious AEs

	*Number of patients (%)*		*Number of patients (%)*
Any AE	31 (100)	Any serious AE	4 (12.9)
Any AE with reasonable possibility of being related to LCIG (drug/device)	30 (96.8)	Any serious AE with reasonable possibility of being related to LCIG (drug/device)	2 (6.5)
	
*AEs occurring in ⩾5% patients by preferred term*		*Serious AEs occurring in any patient by preferred term*	
Incision site pain	**13 (41.9)**	Abdominal pain	**1 (3.2)**
Excessive granulation tissue	**10 (32.3)**	Constipation	1 (3.2)
Constipation	7 (22.6)	Device dislocation	**1 (3.2)**
Diarrhea	6 (19.4)	Device kink	**1 (3.2)**
Fall	6 (19.4)	Disseminated intravascular coagulation	1 (3.2)
Nasopharyngitis	6 (19.4)	Femur fracture	1 (3.2)
Blood homocysteine increased	5 (16.1)	Gastrointestinal perforation	**1 (3.2)**
Dyskinesia	5 (16.1)	Melena	1 (3.2)
Procedural pain	**5 (16.1)**	Pneumonia aspiration	1 (3.2)
Incision site erythema	**4 (12.9)**	Sepsis	1 (3.2)
Anemia	3 (9.7)		
Anxiety	3 (9.7)		
Tinea pedis	3 (9.7)		
Abdominal distension	2 (6.5)		
Abdominal pain	**2 (6.5)**		
Abdominal pain upper	**2 (6.5)**		
Blood pressure decreased	2 (6.5)		
Complication of device insertion	**2 (6.5)**		
Eczema	2 (6.5)		
Epistaxis	2 (6.5)		
Headache	2 (6.5)		
Incision site rash	2 (6.5)		
Insomnia	2 (6.5)		
Musculoskeletal pain	2 (6.5)		
Oropharyngeal pain	2 (6.5)		
Pneumonia aspiration	2 (6.5)		
Pruritis	2 (6.5)		
Pyrexia	2 (6.5)		
Stoma site infection	**2 (6.5)**		
Suture related complication	2 (6.5)		
Toothache	2 (6.5)		
Weight decreased	2 (6.5)		

Abbreviation: LCIG, levodopa–carbidopa intestinal gel.

*N*=31. Gastrointestinal- and gastrointestinal procedure-related AEs are in bold.
