# Supplementary material for: Efficacy and safety of levodopa–carbidopa intestinal gel from a study in Japanese, Taiwanese, and Korean advanced Parkinson’s disease patients
Source: NPJ Parkinsons Dis. 2016 Nov 3;2:16020–. doi: 10.1038/npjparkd.2016.20 (PMC5516619; doi:10.1038/npjparkd.2016.20)
Supplement: Supplementary Table 1 [file npjparkd201620-s1.doc]

Supplemental Table 1. Most Common Anti-parkinsonian Medication Before and Concomitant Medication During the Study

| **Anti-PD Medications Taken Before the Study** | **Number of patients (%)** | **Concomitant Medication During the Study** | **Number of patients (%)** |
| --- | --- | --- | --- |
| Any | 31 (100) | Any | 31 (100) |
| **Reported in ≥ 5 Patients** |  | **Reported in ≥ 5 Patients** |  |
| Sinemet/Menesit | 25 (80.6) | Sodium chloride | 27 (87.1) |
| Entacapone | 15 (48.4) | Midazolam | 26 (83.9) |
| Zonisamine | 12 (38.7) | Lidocaine | 25 (80.6) |
| Amantadine | 10 (32.3) | Cefazolin | 25 (80.6) |
| Ropinirole | 9 (29.0) | Gastrografin | 19 (61.3) |
| Madopar | 8 (25.8) | Hyoscine | 18 (58.1) |
| Pramipexole | 8 (25.8) | Magnesium oxide | 16 (51.6) |
| Rotigotine | 8 (25.8) | Diclofenac | 15 (48.4) |
| Apomorphine | 6 (19.4) | Flumazenil | 15 (48.4) |
| Istradefylline | 6 (19.4) | Loxoprofen | 13 (41.9) |
| Selegiline | 6 (19.4) | Carbohydrates NOS w/potassium chloride/sodium | 12 (38.7) |
|  |  | Domperidone | 12 (38.7) |
|  |  | Mecobalamin | 12 (38.7) |
|  |  | Clonazepam | 11 (35.5) |
|  |  | Ketoprofen | 11 (35.5) |
|  |  | Mosapride | 10 (32.3) |
|  |  | Oxygen | 10 (32.3) |
|  |  | Zolpidem | 10 (32.3) |
|  |  | Dimeticone | 9 (29.0) |

N=31.
