# Supplementary material for: Efficacy and safety of levodopa–carbidopa intestinal gel from a study in Japanese, Taiwanese, and Korean advanced Parkinson’s disease patients
Source: NPJ Parkinsons Dis. 2016 Nov 3;2:16020–. doi: 10.1038/npjparkd.2016.20 (PMC5516619; doi:10.1038/npjparkd.2016.20)
Supplement: Supplementary Table 2 [file npjparkd201620-s2.doc]

Supplemental Table 2. CGI-C and PGI-C Scores

| **Response Scale (Score)** | **CGI-C, n (%)** | **PGI-C, n (%)** |
| --- | --- | --- |
| Very much improved (1) | 9 (31) | 8 (28) |
| Much improved (2) | 15 (52) | 15 (52) |
| Minimally improved (3) | 4 (14) | 4 (14) |
| No change (4) | 1 (3) | 1 (3) |
| Minimally worse (5) | 0 | 1 (3) |
| Much worse (6) | 0 | 0 |
| Very much worse (7) | 0 | 0 |

N=29. The CGI-C and PGI-C are 7-point Likert scales ranging from 1 (very much improved) to 7 (very much worse) and rated by the clinician (CGI-C) or patient (PGI-C). CGI-C = Clinical Global Impression of Change; PGI-C = Patient Global Impression of Change
